# Bi-national survey of Korea and Japan related to the injection site for ultrasound-guided stellate ganglion blocks and anatomic comparisons using cadaver dissection

**DOI:** 10.1371/journal.pone.0232586

**Published:** 2020-05-01

**Authors:** Hyung-Sun Won, Masako Iseki, Satoshi Hagihira, Younhee Kuk, Yeon-Dong Kim, Hyungtae Kim

**Affiliations:** 1 Department of Anatomy, Wonkwang University School of Medicine, Iksan, Korea; 2 Jesaeng-Euise Clinical Anatomy Center, Wonkwang University School of Medicine, Iksan, Korea; 3 Department of Anesthesiology and Pain Medicine, Juntendo University Graduate School of Medicine, Tokyo, Japan; 4 Department of Anesthesiology, Kansai Medical University, Hirakata City, Osaka, Japan; 5 Department of Anesthesiology and Pain Medicine, Wonkwang University School of Medicine, Wonkwang University Hospital, Iksan, Korea; 6 Wonkwang Institute of Science, Wonkwang University School of Medicine, Iksan, Korea; 7 Department of Anesthesiology and Pain Medicine, Asan Medical Center, University of Ulsan College of Medicine, Seoul, Korea; University Magna Graecia of Catanzaro, ITALY

## Abstract

The aims of this study were to investigate the current clinical practice of ultrasound (US)-guided stellate ganglion block (SGB) using a bi-national survey of Korea and Japan, and to clarify the anatomical relation of the cervical sympathetic trunk with the prevertebral fascia at the level of cervical vertebrae. The current clinical practice of US-guided SGB in Korea and Japan was investigated using an Internet survey, which received 206 (10.2%) replies from Korea and 97 (8.8%) replies from Japan. The survey questionnaire addressed the actual clinical practice for US-guided SGB, including where the tip of the injection needle is placed. Additionally, 16 half necks of 8 embalmed cadavers were used in an anatomical study. An in-plane needle approach technique and administering 5 ml of local anesthetic were preferred in both countries. However, the type of local anesthetic differed, being lidocaine in Korea and mepivacaine in Japan. The final position of the needle tip also clearly differed in an US image, being predominantly positioned above the prevertebral fascia in Korea (39.3%) and under the prevertebral fascia in Japan (59.8%). In all of the anatomic dissections, the cervical sympathetic trunk was over the prevertebral fascia at the level of the sixth vertebra and under the prevertebral fascia at the level of the seventh vertebra. These results are expected to improve the knowledge on the current clinical practice and to suggest future studies.

## Introduction

Stellate ganglion block (SGB; also known as cervical sympathetic block) is commonly utilized in diagnostic, prognostic, and therapeutic interventions for sympathetically mediated pain [1], and vascular insufficiency of the upper extremities, such as phantom pain, zoster-associated pain, cancer pain, orofacial pain, and vascular headache [2].

Various techniques for performing an SGB have been developed and reported. From a historical perspective, the blind technique has been the mainstay of treatment at the ganglion level [3,4]. However, due to the uncertainty of drug injections with the blind technique, complications, such as hoarseness, seizures, dyspnea, and hematoma formation, were possible [5]. Ineffectiveness associated with unintentional injections causing injury to a structure was also a main concern. Efforts to improve the safety of the injection process have resulted in the techniques for SGB evolving over time. Approaches were used in the blind technique, with this subsequently changing through the use of guidance from fluoroscopy and computed tomography, and recently the ultrasound (US)-guided approach [6–9]. In particular, guidance from US makes it easy for the pain physician to place the needle tip in the ideal position, which results in a more effective and precise sympathetic block even when only a small volume of local anesthetic is injected [10]. US-guided SGB may also eventually improve the safety of the procedure itself by minimizing the risk of structural damage. An accurate knowledge of anatomy is also essential to performing safe and effective blockades, and so the exact positions of the cervical sympathetic trunk and stellate ganglion have always been of considerable interest to interventional pain physicians. However, topographic descriptions of the cervical sympathetic trunk have been reported differently according to the literature [11–13].

The use of various (and ambiguous) types and volumes of drugs, methods, and final positions of the needle tip during US-guided SGB in clinical practice has also been reported in the literature. In addition, there is a lack of research on the actual practice of US-guided SGB. This study was performed to report on the current practice of US-guided SGB in Korea and Japan through a questionnaire survey, and to confirm the actual relationship between the cervical sympathetic trunk and the prevertebral fascia in cadaver dissections.

## Materials and methods

All of the study including both of the internet survey and anatomic dissection were approved by the institutional review board of Wonkwang University Hospital (IRB ID No. WKUH 2018-05-005).

### Internet survey

Practical information about how US-guided SGB is performed currently in Korea and Japan was collected via an Internet survey. E-mails containing questionnaires for the survey were sent to 2,023 and 1,038 pain clinicians who were registered members of the Korean Society of Anesthesiologists and the Japan Society of Pain Clinicians. All participants were recruited on the condition that they were willing to participate in various research methods, such as a survey, and therefore, the acquisition of the consent form was waived.

**Fig 1:**
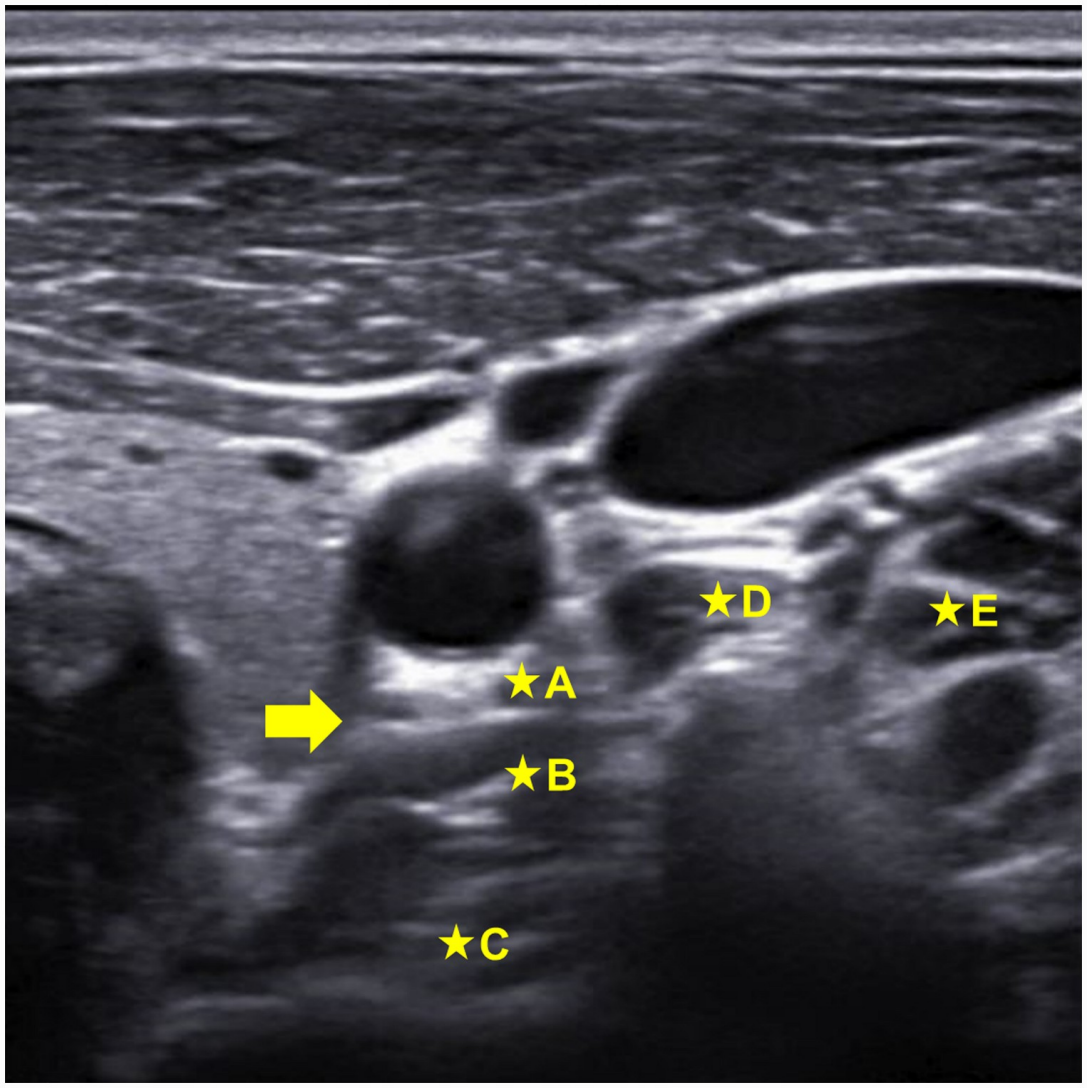
US image used in the survey of the final position of the needle tip. The arrow indicates the prevertebral fascia (PVF), which has not been included in the figure for survey. A, above the PVF; B, under the PVF; C, in the longus colli muscle; D, in the longus capitis muscle; E, in the anterior scalene muscle.

The contents of the questionnaire survey were as follows: (1) the clinical career of the responder relevant to SGB, (1) the frequency of using US during SGB, (3) the types and volumes of local anesthetics used, (4) if using US, whether an in-plane or out-of-plane needle approach technique was preferred, and (5) the final position of the needle tip, as indicated on a US image (Fig 1). The results for the two countries were analyzed and compared using statistical tools.

### Anatomical dissection

After the survey, 16 half necks of 8 Korean cadavers embalmed by perfusion with our own fixation mixture, including the 8% formaldehyde solution, ethanol, phenol, and glycerin, were used in an anatomical study (3 males and 5 females; mean age at death of 82.4 years, age range at death of 70–90 years). None of the cadavers exhibited any evidence of gross pathology, previous surgical procedures, or traumatic lesions of the neck. All cadavers stored in Jesaeng-Euise Institute, Wonkwang University School of Medicine are qualified as the materials for education and research, according to the domestic law that the cadavers must be donated and managed under the informed consent and ethical process. All of the study procedures were performed in accordance with the Declaration of Helsinki.

The investing fascia was first exposed after removing the skin, subcutaneous tissue, and platysma of the anterior neck. The infrahyoid muscles were then fully exposed after reflecting the investing fascia and sternocleidomastoid muscle. All of the specimens were then sectioned midsagittally under the prevertebral fascia using a sharp blade. Finally, the prevertebral fascia was laterally reflected and carefully separated from the carotid sheath, and the exact positions of the cervical sympathetic trunk and ganglion relative to the prevertebral fascia were observed at the levels of the sixth and seventh vertebrae (C6 and C7, respectively).

### Statistics

All statistical analyses were conducted using SPSS (version 20.0, IBM SPSS, USA). Student’s *t*-test or the chi-square test was used to compare between the numerical or categorical data of two groups, and a cutoff probability level of 0.05 was chosen. All variables are described as number or percentage values.

## Results

**Fig 2:**
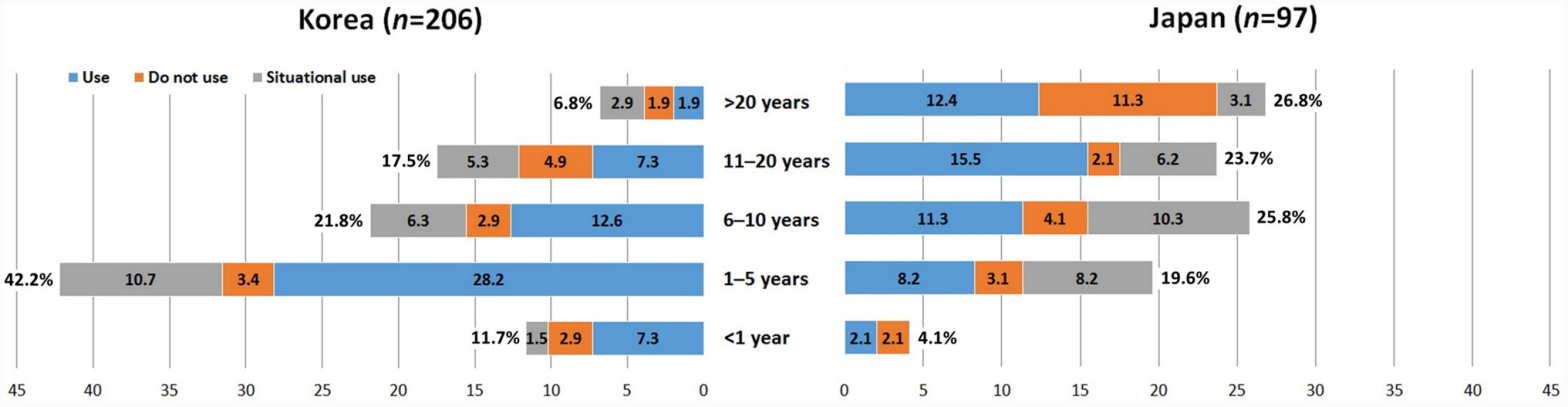
Comparisons of physicians using US during SGB with respect to clinical experience in Korea and Japan.

**Fig 3:**
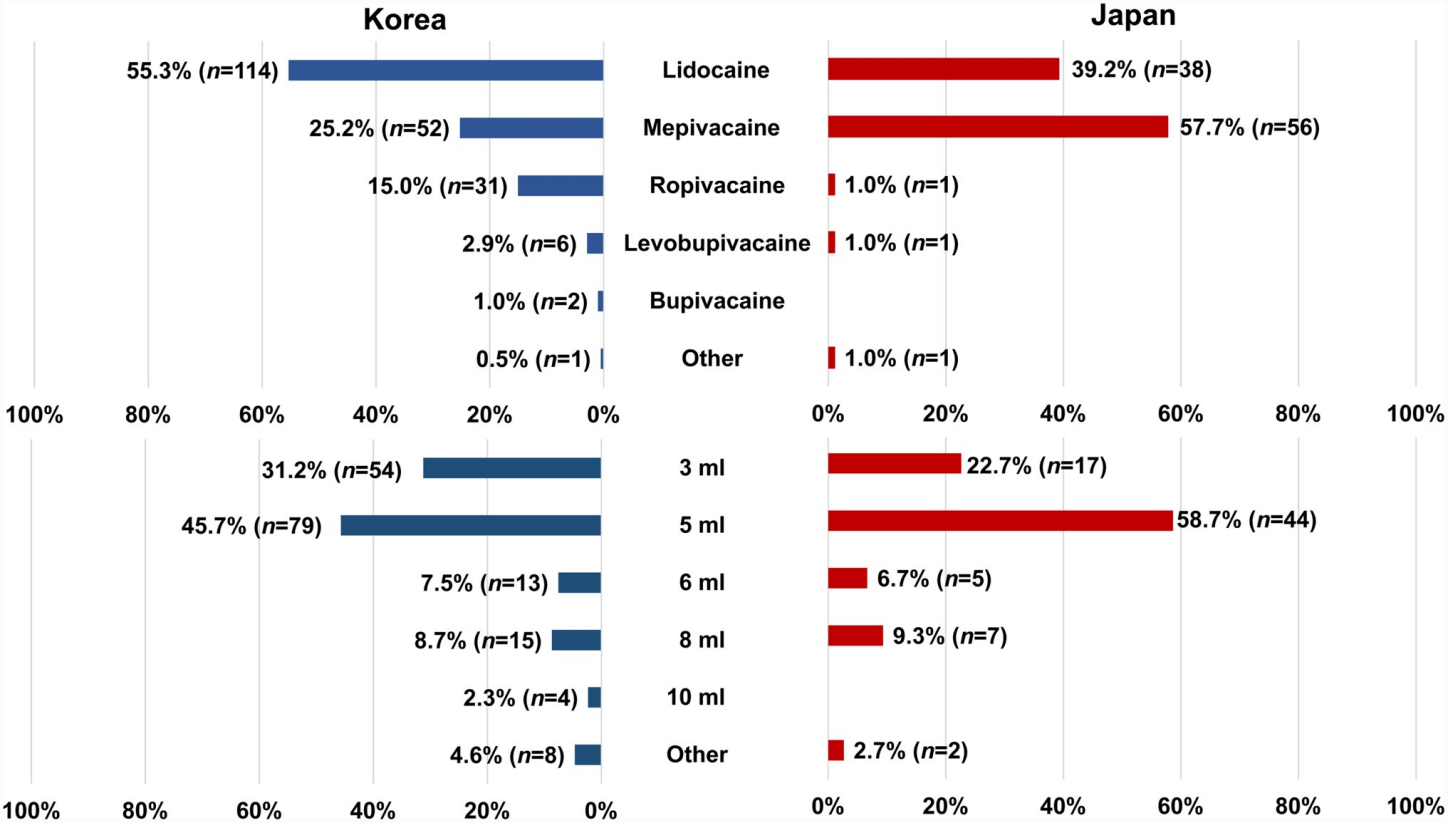
Comparison of types and volumes of local anesthetics used for SGB in Korea and Japan.

**Fig 4:**
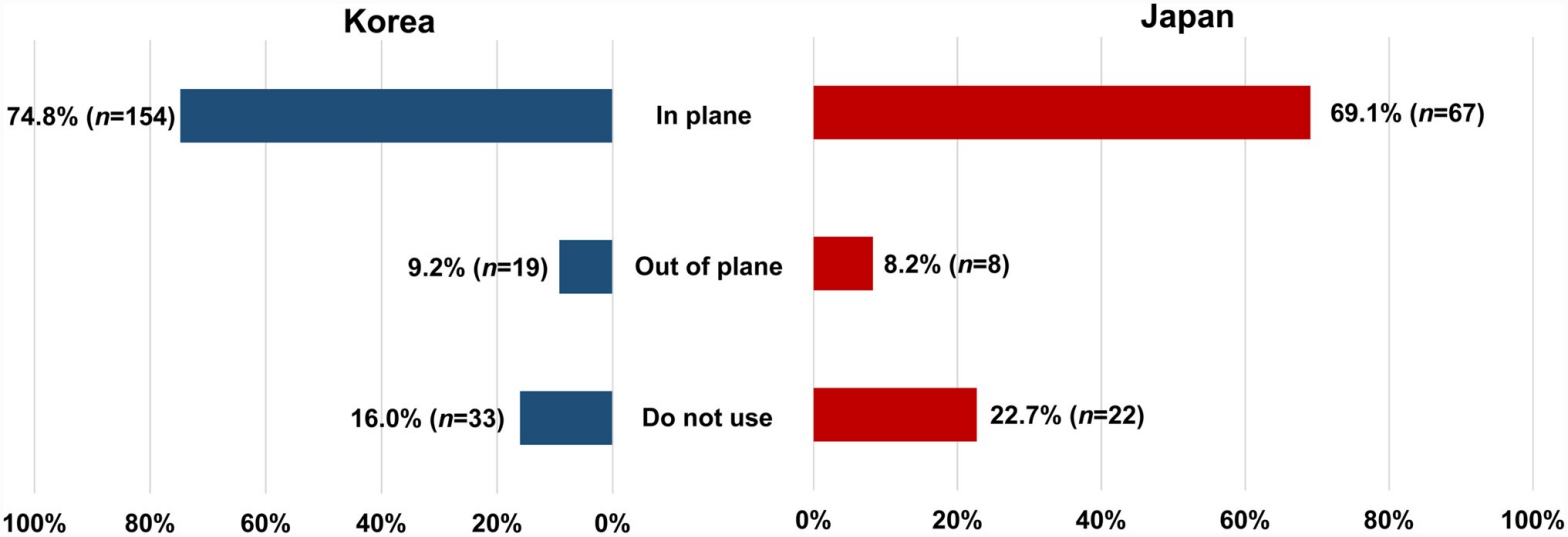
Comparison of preferred types of needle approach for SGB in Korea and Japan.

**Fig 5:**
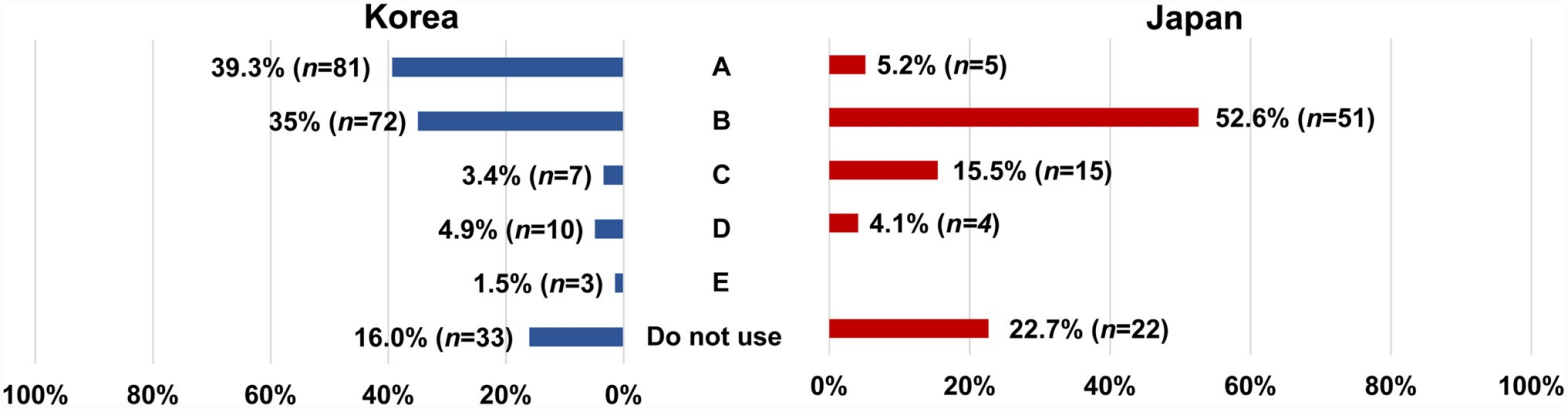
Comparison of final positions of the needle tip for US-guided SGB in Korea and Japan.

Responses to the survey questionnaire on the current clinical practice of US-guided SGB were received from 303 pain physicians in Korea (*n* = 206) and Japan (*n* = 97). The rate of response was 10.2% from Korea and 8.8% from Japan. The frequency of using US during SGB in each country differed, being 84.0% (*n* = 173) in Korea and 77.3% (*n* = 75) in Japan (*p* = 0.02, Fig 2). The most common type of local anesthetic used for SGB was lidocaine in Korea (55.3%, *n* = 114) and mepivacaine in Japan (57.7%, *n* = 56) (Fig 3). There was no significant difference in the volume of local anesthetics used for SGB between the two countries (Fig 3), with 5 mL being the most common. Regarding the type of needle approach during US-guided SGB, respondents from both countries preferred the in-plane technique, and the method chosen did not differ significantly between Korean and Japan (Fig 4). The final position of the needle tip when using US-guided SGB differed significantly between the two countries with 81 of the Korean respondents (39.8%) preferring the tip to be above the prevertebral fascia, while 51 of the Japanese responders (52.6%) preferred the tip to be under the prevertebral fascia (*p* <0.001, Fig 5).

**Fig 6:**
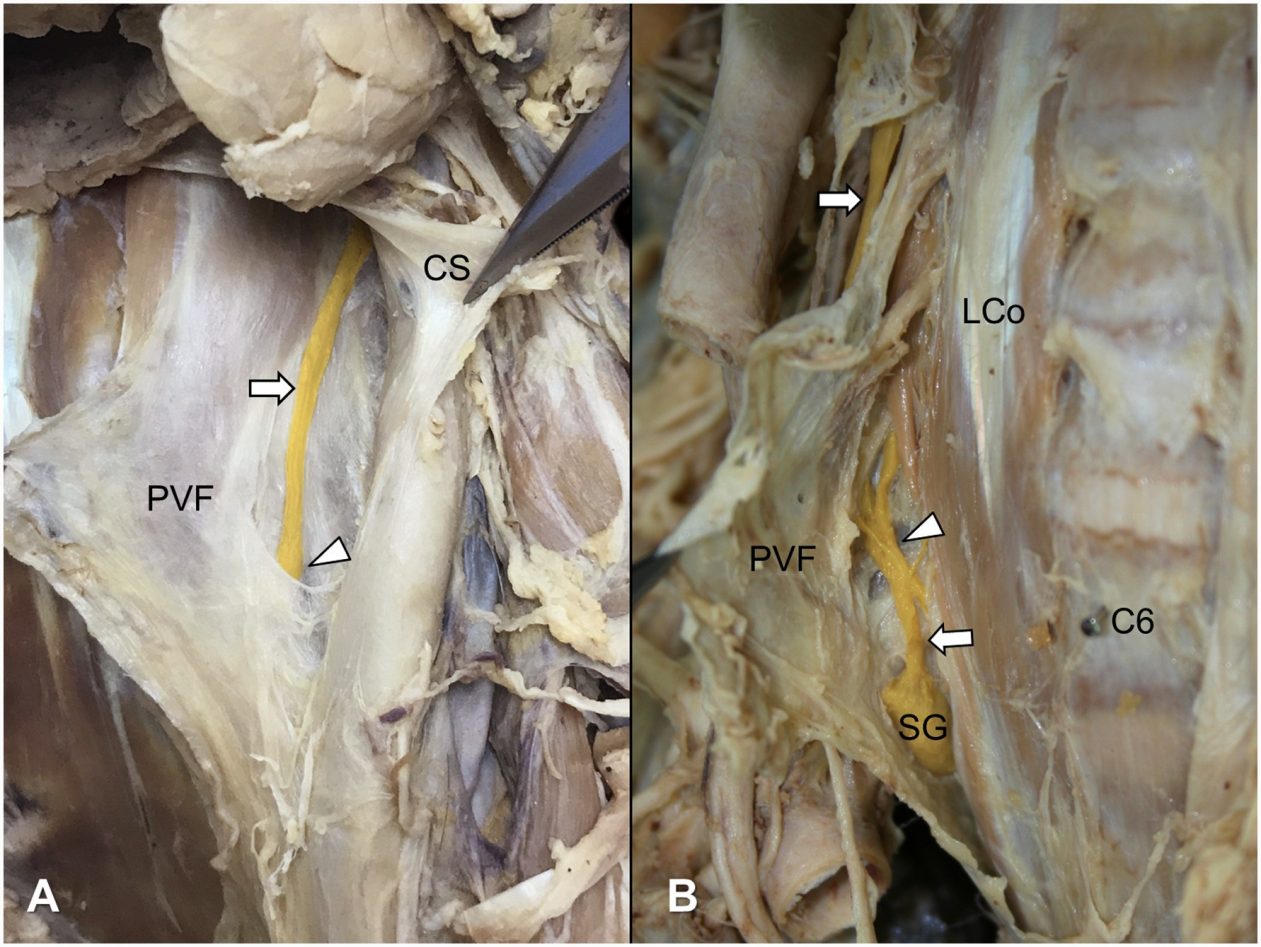
The topographical relationships between the cervical sympathetic trunk and prevertebral fascia above (A) and below (B) the C6 vertebra. Arrowheads and arrows indicate the middle cervical ganglion and cervical sympathetic trunk, respectively. CS, carotid sheath; PVF, prevertebral fascia; LCo, longus colli muscle; C6, sixth cervical vertebra; SG, stellate ganglion.

To confirm the location of the cervical sympathetic trunk with respect to the levels of the cervical vertebra and the prevertebral fascia, we dissected both sides of the neck of eight cadavers. All of the cervical sympathetic trunks passed over the prevertebral fascia at the C6 vertebral level (Fig 6A), while they passed under the fascia at the C7 vertebral level (Fig 6B). The middle cervical ganglion was generally found in the region where the sympathetic trunk penetrated the prevertebral fascia (Fig 6).

## Discussion

The most popular technique for SGB is an anterior paratracheal approach at the C6 vertebral level, based on the assumption that the drug will spread caudally to the stellate ganglion [7]. When the blind technique is used for SGB, the cricoid cartilage is regarded as a remarkable landmark of the C6 vertebral level. After palpating the anterior tubercle of the transverse process of the C6 vertebra and confirming no aspiration of blood, the drug is injected around it. This technique involves directing the needle toward the bone and then withdrawing it while injecting the solution, which means that the needle tip is located in the longus colli muscle. However, the final position of the needle and the spread of the local anesthetic agent are unpredictable with the blind technique, which may result in unintended serious injections (vascular, subdural, or intrathecal) [14–16].

The fluoroscopy-guided technique was introduced to monitor the spread of a contrast agent and thereby rule out intravascular, epidural, and intrathecal injections [17]. However, this technique also cannot reveal the soft tissues traversed along the needle path, and the presence of major anatomic vascular variations has been highlighted as a risk factor. In contrast to the blind and fluoroscopy-guided techniques, US can help the physician to identify the cervical sympathetic ganglion and the prevertebral fascia over the longus colli muscle in real-time imaging, confirm the spread of drugs, prevent unintentional puncturing of blood vessels, and reduce the volume of local anesthetic required to achieve reliable blockade thanks to it being deposited in an exact position.

The key point for successful SGB is to deposit a small volume of local anesthetic around the target area, reducing the incidence of adverse effects. The present study found that despite being able to accurately confirm the positions of relevant structures of the neck in an US image, the responders reported a variety of positions for the needle tip. This can be attributed to various reports in the literature related to the position of the needle tip in US images, such as above or under the prevertebral fascia, or even in the longus colli muscle [18–20]. It is also assumed that the current practice of US-guided SGB represents a phase of transition from the blind technique to the US-guided technique designed to inject the drug into the longus colli muscle [11,21]. Regarding the target of injections, the caudal spread of solution to the stellate ganglion itself will be more extensive for a subfascial than a suprafascial injection [22]. However, the exact location should be reassessed since these results were not based on anatomic verification. Unintentional recurrent laryngeal nerve block from overdosage and intravascular injection from insufficient anatomic information could cause hoarseness and seizure, respectively. US-guided SGB could be performed through the prescanning for obtaining accurate information, which could reduce the volume of injected material.

The stellate ganglion, which is formed by the fusion of the inferior cervical and first thoracic ganglions, has been described as extending from the level of the head of the first rib to the inferior border of the transverse process of the C7 vertebra, with it lying medial or sometimes posterior to the vertebral artery immediately adjacent to the dome of pleura [23–25]. In the present study, in most cases either the middle cervical sympathetic ganglion or no ganglion was observed at the C6 vertebral level, and the inferior cervical sympathetic or stellate ganglion started to be observed at or below the C7 vertebral level.

The prevertebral layer of the cervical fascia (prevertebral fascia) covers the anterior vertebral muscles (i.e., longus capitis and longus colli muscles), and extends laterally over the anterior, middle scalene muscles and levator scapulae to form a fascial floor for the posterior triangle of the neck [26]. The prevertebral fascia is connected anteriorly to the carotid sheath by a relatively loose tissue containing the cervical sympathetic trunk [27]. The entire anterior rami of the cervical nerves are initially deep to the prevertebral fascia. The dorsal scapular, long thoracic, and phrenic nerves retain this position throughout their course in the neck [26]. In the present study, the observation of the prevertebral fascia was consistent with the aforementioned descriptions, except for the relation with the cervical sympathetic trunk. The cervical sympathetic trunk passed superficial to the prevertebral fascia at the C6 vertebral level (Fig 6A) and deep to the fascia at the C7 vertebral level (Fig 6B). In addition, the prevertebral fascia could be obviously distinguished by a loose connective tissue from the carotid sheath enclosing the common carotid artery, internal jugular vein, and vagus nerve. A previous study using a low-volume (0.2 mL) dye injection reported that the cervical sympathetic trunk was strongly stained deep to the prevertebral fascia at the C6 vertebral level [21]. However, the sympathetic trunk seems to be on the fascia covering the longus colli muscle in the suggested figure of that study.

Based on the anatomic information obtained in the present study, injecting above the prevertebral fascia seems to be more effective for performing a cervical sympathetic block at the C6 vertebral level using US even when only a small volume of anesthetic is injected. However, limitation of this study is the relative low response rate to the survey which does not fully reflect the overall clinical practice in both countries. The other is that it does not provide suggestions for the optimal quality (volume and concentration) of local anesthetics used for SGB in different cervical regions due to its observational nature, limited number of cadaveric dissections, and comparative clinical study design using US. Under the US guidance, clinical effectiveness associated with different positions of the needle tip relative to the prevertebral fascia and different types of local anesthetics should be investigated in future studies. Furthermore, studies are also required to investigate the effects of the volume of the injection with a large number of cases that include anatomic variations based on our study.

In conclusion, this is the first study on the current practice of US-guided SGB showing different forms of clinical practices in Japan and Korea. This study showed differences between the two countries in the final needle tip position during SGB related to anatomy. It also investigated the topographic relationship between the cervical sympathetic chain and the prevertebral fascia in the cervical spine. Our results suggest that the current common practice for blockage at the C6 vertebral level is a middle cervical ganglion block rather than an SGB. Injecting under the prevertebral fascia is a more anatomically correct position when performing a sympathetic block at the C7 vertebral level, and therefore, it could be described as a real SGB. However, when the physicians try a real SGB at the C7 vertebral level, special care should be taken not to spread the local anesthetic into the vertebral artery or phrenic nerve.

## Supporting information

S1 File (DOCX)

S2 File (DOCX)

S3 File (DOCX)
