# Supplementary material for: Bi-national survey of Korea and Japan related to the injection site for ultrasound-guided stellate ganglion blocks and anatomic comparisons using cadaver dissection
Source: PLoS One. 2020 May 1;15(5):e0232586. doi: 10.1371/journal.pone.0232586 (PMC7194360; doi:10.1371/journal.pone.0232586)
Supplement: S1 File — (DOCX) [file pone.0232586.s001.docx]

**Survey of the clinical practice on ultrasound guided cervical sympathetic block (Stellate ganglion block; SGB)**

Dear, physicians.

This survey is on the current clinical practice on the cervical sympathetic block (What is called stellate ganglion block: SGB) in your clinic.

Please reply on your practice.

Thank you for your help.

**1. How long is your clinical experience on your clinical practice relater to pain management?**

1. Under 1 year

2. 1-5 years

3. 6-10 years

4. 11-20 years

5. Over 20 years

**2. Do you use ultrasound when you perform SGB?**

1. Always use ultrasound

2. Do not use ultrasound

3. Sometime use ultrasound, depending on the situations

**3. When you perform SGB, What kind of local anesthetics do you use ?**

1. Lidocaine

2. Mepivacaine

3. Ropivacaine

4. Levo-bupivacaine

5. Bupivacaine

6. etc

**4. How much volume of local anesthetics when you perform SGB?**

1. 3ml

2. 5ml

3. 6ml

4. 8ml

5. 10ml

6. etc

**5. On the needle approach technique, which method do you use? Please select one.**

1. In-plane technique

2. Out-of-plane technique

3. etc

**6. This is the image of ultrasound guided SGB. Where is your need tip when you perform SGB in your practice? Please select one.**


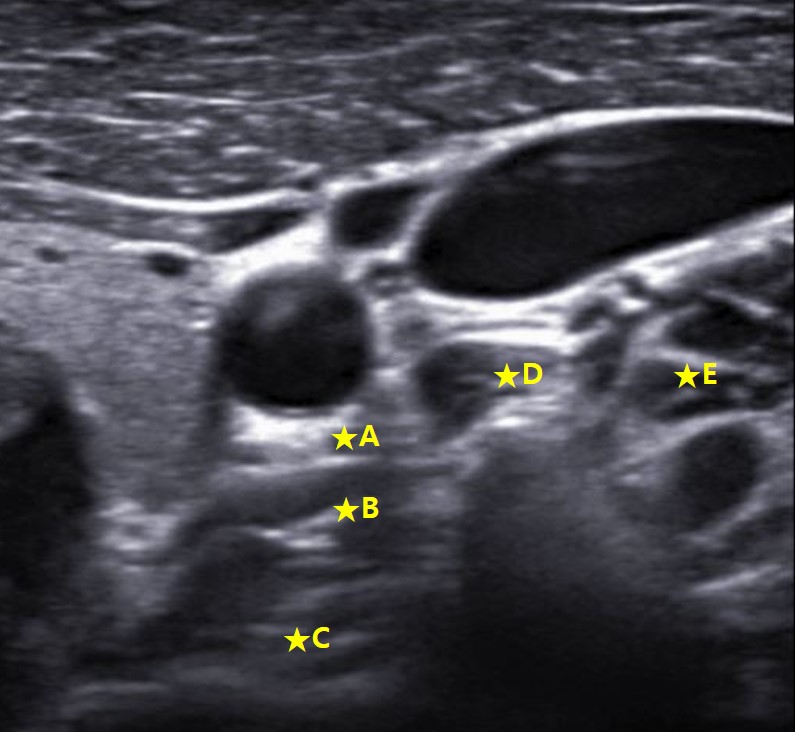


1. A

2. B

3. C

4. D

5. E
