# Supplementary material for: Bi-national survey of Korea and Japan related to the injection site for ultrasound-guided stellate ganglion blocks and anatomic comparisons using cadaver dissection
Source: PLoS One. 2020 May 1;15(5):e0232586. doi: 10.1371/journal.pone.0232586 (PMC7194360; doi:10.1371/journal.pone.0232586)
Supplement: S2 File — (DOCX) [file pone.0232586.s002.docx]

**超音波ガイド下 星状神経節ブロック(Stellate ganglion block; SGB)の臨床施行現況調査研究**

本設問は**頸椎部星状神経節ブロック** (Stellate Ganglion Block: SGB)の臨床施行現況に関する調査研究です。

現在診療現場で行なってる事項についてお答えください。

**1. 先生の専攻科目をお教えください。**

1. 麻酔科

2. リハビリテーション科

3. 脳神経外科

4. 整形外科

5. 総合診療科

その他:

**2. 現在までのペインクリニックの経歴をお教えください。**

1.１年未満

2. 1-5年

3. 6-10年

4. 11-20년

5. 20年以上

**3. SGBの際に超音波は使用されていますか？**

1. 超音波をいつも使っている

2. 超音波は使ってない

3. 状況に応じて使っている

**4. SGBの際に使用されている局所麻酔薬はどれでしょうか。**

1. Lidocaine

2. Mepivacaine

3. Ropivacaine

4. Levo-bupivacaine

5. Bupivacaine

その他:

**5. 超音波ガイド下** **SGBの際に注入する局所麻酔薬の用量は？**

1. 3ml

2. 5ml

3. 6ml

4. 8ml

5. 10ml

その他:

**6. 超音波ガイド下** **SGBの際に使用する針のアプローチ法をお選びください。**

1. In-plane technique（平行法）

2. Out-of-plane technique （交差法）

その他:

**7. 超音波ガイド下** **SGBの際に局所麻酔薬を注入する針先の位置は写真のどの部位になりますか？**


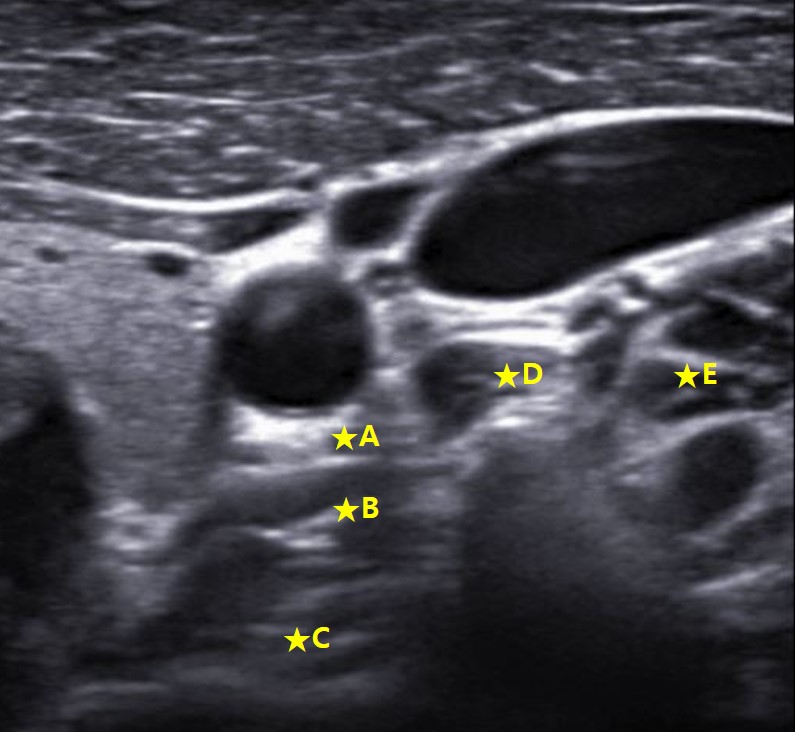


1. A

2. B

3. C

4. D

5. E

ご協力いただき誠にありがとうございます。

ご回答頂いたアンケート調査の内容を精査し、今後皆様に共有出来るよう致します。
