# Supplementary material for: Bi-national survey of Korea and Japan related to the injection site for ultrasound-guided stellate ganglion blocks and anatomic comparisons using cadaver dissection
Source: PLoS One. 2020 May 1;15(5):e0232586. doi: 10.1371/journal.pone.0232586 (PMC7194360; doi:10.1371/journal.pone.0232586)
Supplement: S3 File — (DOCX) [file pone.0232586.s003.docx]

**초음파 유도하 경추부성상신경 차단술(Stellate ganglion block)의 임상시행 현황**

**경추부 성상신경 차단술(Stellate Ganglion Block: SGB)의 임상 시행 현황에 관한 설문 연구입니다.**

**현재 진료에서 시행하고 있는 사항에 대해 설문에 응해 주시기를 요청 드립니다.**

**1. 현재 통증치료의 경력은 얼마나 되는지 선택해주세요**

1. 1년미만

2. 1-5년

3. 5-10년

4. 10년이상

5. 20년이상

**2. SGB 시행시 초음파 사용 여부를 답해주세요**

1. 초음파를 항상 사용

2. 초음파 사용하지 않고 시행

3. 초음파를 상황에 따라 이용하여 시행

**3. SGB 시행시 사용하는 국소마취제의 종류를 선택하세요**

1. Lidocaine

2. Mepivacaine

3. Ropivacaine

4. Levo-bupivacaine

5. Bupivacaine

**4. 초음파 유도하 SGB 시행시 이용하는 국소마취제의 용량을 선택해주세요**

1. 3ml

2. 5ml

3. 6ml

4. 8ml

5. 10ml

기타:

6. 초음파 유도하 SGB 시행시 다음의 초음파 그림을 보고 평소 바늘 끝(Needle tip)의 최종 위치를 어디에 위치하는지 선택해주세요

1. A

2. B

3. C

4. D

5. E

기타:
